# The Complete Genome Sequence of *Bacillus toyonensis* Cbmb3 with Polyvinyl Chloride-Degrading Properties

**DOI:** 10.3390/jox14010018

**Published:** 2024-02-26

**Authors:** Dandan Wang, Hong Yu, Xinbei Liu, Li Sun, Xijian Liu, Ruilong Hu, Chao Wang, Yuping Zhuge, Zhihong Xie

**Affiliations:** National Engineering Research Center for Efficient Utilization of Soil and Fertilizer Resources, College of Resources and Environment, Shandong Agricultural University, Taian 271018, China; wangddnan@163.com (D.W.); 2022120248@sdau.edu.cn (H.Y.); 18854808281@163.com (X.L.); 2021010045@sdau.edu.cn (L.S.); 2023120232@sdau.edu.cn (X.L.); 2023120228@sdau.edu.cn (R.H.); chaow210@163.com (C.W.); zhugeyp@sdau.edu.cn (Y.Z.)

**Keywords:** polyvinyl chloride, biodegradation, *Bacillus toyonensis*, environmental microorganism, complete genome sequence

## Abstract

The accumulation of high amounts of plastic waste in the environment has raised ecological and health concerns, particularly in croplands, and biological degradation presents a promising approach for the sustainable treatment of this issue. In this study, a polyvinyl chloride (PVC)-degrading bacterium was isolated from farmland soil samples attached to waste plastic, utilizing PVC as the sole carbon source. The circular chromosome of the strain Cbmb3, with a length of 5,768,926 bp, was subsequently sequenced. The average GC content was determined to be 35.45%, and a total of 5835 open reading frames were identified. The strain Cbmb3 was designated as *Bacillus toyonensis* based on phylogenomic analyses and genomic characteristics. The bioinformatic analysis of the Cbmb3 genome revealed putative genes encoding essential enzymes involved in PVC degradation. Additionally, the potential genomic characteristics associated with phytoprobiotic effects, such as the synthesis of indole acetic acid and secondary metabolite synthesis, were also revealed. Overall, the present study provides the first complete genome of *Bacillus toyonensis* with PVC-degrading properties, suggesting that Cbmb3 is a potential strain for PVC bioremediation and application.

## 1. Introduction

The extensive utilization of plastic has resulted in the gradual accumulation of plastic waste in the environment, leading to a severe ecological crisis [1]. The extensive utilization of plastics in agricultural practices, encompassing plastic films, irrigation pipes, and nets, coupled with the inadequate rates of plastic recycling, leads to the excessive accumulation of plastic waste [2]. This issue poses long-term threats to the sustainability of farmland ecosystems and serves as a primary contributor to terrestrial microplastic (MP) pollution [3]. Polyvinyl chloride (PVC) is a widely utilized plastic polymer with the chemical formula [C_2_H_3_Cl]n and is insoluble in water and alcohol, thereby resulting in a prolonged degradation process when introduced into the environment [4]. Additionally, the weathering degradation of plastics leads to embrittlement and microcracking, resulting in the generation of MPs and nanoplastics that persist in the environment [5]. For example, in China, farmland soil samples were found to be universally contaminated with microplastics, particularly in open-field cultivation soils (4308 items/kg), with a PVC content of 40.9% [6,7].

The conventional methods to dispose of PVC include incineration, landfilling, and recycling, but these methods have several limitations, like low efficacy, high costs, and potential secondary pollution [4]. Biodegradation, particularly microbial degradation, is gaining increasing attention due to its economic and environmental benefits [8]. This method has the potential to effectively mitigate the negative impact of PVC in an environmentally friendly manner while simultaneously alleviating stress and promoting plant growth [9]. Several studies have proposed the possibility of PVC biodegradation by several fungi or bacteria isolated from diverse environments, including *Klebsiella* [10], *Pseudomonas*, *Bacillus* [11], *Malassezia*, *Aspergillus* [12], and so on. Additionally, the proposed biodegradation pathway as well as the potential genes and proteins involved in PVC degradation were identified through comprehensive genomic, transcriptomic, proteomic, and metabolomic analyses. In *Klebsiella* sp. EMBL-1, PVC underwent depolymerization into lower-molecular-weight polymers via catalysis by catalase–peroxidase. Subsequently, the production of -C=C- bonds and hydroxyl groups was further promoted by oxidation. After that, a series of enzymatic reactions, potentially mediated by laccase, dioxygenase, and other enzymes, ultimately led to degradation into small organic molecules or fatty acids to support the growth of the strain [10]. However, the current germplasm resources for effective PVC degradation are insufficient and lag behind those for the biodegradation of polyethylene (PE) and other plastic polymers. Moreover, there is a scarcity of reports on the isolation, identification, and comprehensive genetic analysis of soil-based PVC-degrading strains. The investigation of the impacts of plastic pollution on terrestrial ecosystems is a paramount concern in environmental research [13]. Therefore, it is imperative to isolate and screen more efficient functional microbes and explore the metabolic pathway for PVC degradation.

In the present study, a PVC-degrading strain, Cbmb3, is reported; it was isolated from plastic-attached soil samples that were collected from saline–alkaline farmland and identified as the species *Bacillus toyonensis*. To further study the functional genes and specific mechanisms of Cbmb3, whole-genome sequencing was performed, and the potential genetic basis for PVC degradation in the Cbmb3 genome was explored. *Bacillus* species are important plant growth promoters, and it has been reported that some bacilli could enhance plant growth while simultaneously degrading microplastics [14]. Therefore, the strain Cbmb3 was also explored for its potential for PVC plant growth promotion and soilborne disease suppression. Overall, *Bacillus toyonensis* Cbmb3 could be a new agent for the bioremediation and biodegradation of PVC.

## 2. Materials and Methods

### 2.1. Strain and Medium

The strain Cbmb3 was isolated from a saline–alkaline soil using a mineral salt medium (MSM) with PVC as the sole carbon source. The soil was attached to waste plastics collected from an agricultural field in Dongying County, Shandong Province, China. One gram of soil was diluted in 10 mL of sterile water, followed by spreading 100 μL of the soil supernatant onto MSM agar plates. The Petri dishes were incubated at 35 °C, and the growth of microorganisms was monitored every 12 h.

The growth curves were assessed in Luria–Bertani medium (LB) with different concentrations of NaCl and pH values. A single colony of Cbmb3 was inoculated into liquid LB medium and incubated at 35 °C until the optical density at OD_600_ reached 1.0. Subsequently, 1 mL of this culture was transferred into 100 mL of liquid LB medium with varying concentrations of NaCl and pH levels, followed by incubation at 35 °C and 180 rpm. Afterward, the samples were analyzed using a spectrophotometer to measure the optical density at OD_600_.

MSM: K_2_HPO_4_ 1.6 g/L, (NH_4_)_2_SO_4_ 1 g/L, KH_2_PO_4_ 0.2 g/L, MgSO_4_·7H_2_O 0.1 g/L, CaCl_2_ 0.05 g/L, NaCl 0.02 g/L, and FeSO_4_·2H_2_O 0.02 g/L, containing PVC 1 g/L as the sole carbon source [15], and, if needed, 2.5% agar for solidification.

LB: peptone 10 g/L, yeast extract 5 g/L, NaCl 10 g/L, and, if needed, 2.5% agar for solidification.

The PVC powder was purchased from Shanghai Macklin Biochemical Technology Co., Ltd.; the K-value is 72-71, the particle size is 0.25–0.5 mm, and the density is 1.4 g/mL at 25 °C.

### 2.2. Genome Sequencing and Annotation

The Cbmb3 genome was sequenced by Oxford Nanopore Technologies at the Beijing Genomics Institute (BGI, Shenzhen, China). The integrity and purity of the samples were assessed using a NanoDrop ND-2000 spectrophotometer and agarose gel electrophoresis, and then library construction was performed. The bioinformatics analysis proceeded after data filtering; the raw reads were filtered to remove low-quality data using SOAPnuke (Version: 1.5.6) and Porechop (Version: 0.2.4) to build scaffolds and contigs [16]. The genome sequence was assembled using Canu (Version: v1.5) and GATK (Version: v1.6-13) [17,18]. The complete genome sequence was obtained through rectification using Pilon (Version: 1.23) and Unicycler (Version: 0.4.9) [19,20]. The complete genome data were deposited in NCBI GenBank under the BioProject number PRJNA992983, and the circular map was generated using CGView [21].

### 2.3. Phylogenetic Analysis

The preliminary identification of Cbmb3 was based on the 16S rRNA gene (accession number OR826673). First, 16S rRNA sequences of closely related species were collected from the EzBioCloud database [22] and utilized for constructing the phylogenetic tree using maximum-likelihood analysis in MEGA-X [23]. Then, the average nucleotide identity (ANI) and in silico DNA-DNA hybridization (DDH) were applied to evaluate the taxonomic identity using the genome assemblies to further explore the taxonomic position of Cbmb3. The ANI was calculated using JSpecies (Version: 1.2.1), and in silico DDH was calculated using the genome-to-genome distance calculator (GGDC) (Version: 2.1) [24,25].

### 2.4. Genome Component and Gene Annotation

After obtaining a genomic sequence, the analysis of the functional elements’ distribution proceeded. The genome sequence was annotated using Glimmer (Version: 3.02) and RAST (the Rapid Annotations using Subsystems Technology) [26,27]. Non-coding RNA (ncRNA) was predicted using RNAmmer (Version: 1.2), tRNAscan-SE (Version: 1.3.1), and Rfam (Version: 9.1) [28,29,30]. Tandem repeats (TRs) were predicted using Tandem Repeats Finder (Version: 4.04) [31]. The prophage regions were predicted using the PHAge Search Tool—Enhanced Release (PHASTER) [32]. The genomic islands were predicted using IslandViewer 4 [33]. CRISPRs were predicted using CRISPRCasFinder (Version: 4.2.2).

Functional annotation was accomplished through the analysis of protein sequences. Carbohydrate-Active enZYmes (CAZy) was predicted using the Carbohydrate-Active enZYmes Database [34]. The Gene Ontology (GO), Clusters of Orthologous Groups (COG), and Kyoto Encyclopedia of Genes and Genomes (KEGG) databases were used for general functional annotations [35,36,37]. The gene cluster that was related to secondary metabolism was identified using antiSMASH (Version: 7.0.0) [38].

### 2.5. Biodegradation Characterization

The PVC-degrading ability of the strain Cbmb3 was first demonstrated on the MSM agar medium by utilizing PVC powder as the sole carbon source. A single colony of Cbmb3 was inoculated onto the MSM agar medium, followed by incubation at 35 °C for 3 days, and then the agar plate was flooded with an iodine–boric acid solution to observe the development of clear zones around the colony.

Iodine–boric acid solution: KI-I_2_ (25 + 12.7 g/L)/boric acid (40 g/L),1:5 (*v/v*). The color reaction is primarily attributed to the presence of embedded iodine within the polymer matrix. However, when PVC undergoes degradation, its binding with iodine is lost, resulting in a lack of coloration and leading to the formation of a transparent zone surrounding the colony.

The PVC biodegradation assay was further performed in 1 L flasks containing 250 mL of MSM, where 3 cm^2^ (11–12 μm thickness) PVC pieces served as the sole carbon source [11]. The pre-cultured Cbmb3 strain was inoculated at an OD_600_ of 1.0, and then 2.5 mL of the suspension was added to the MSM and incubated at 35 °C and 180 rpm for 30 days. The washed PVC pieces were dried in an air-blower-driven drying closet for 24 h until reaching a constant weight. Subsequently, the weight loss was determined by calculating the percentage relative to the initial gravimetric weight.

### 2.6. Plant-Growth-Promoting Attributes

IAA production was studied according to Salkowski’s method [39]. A single colony of Cbmb3 was inoculated into liquid LB medium and incubated at 35 °C until the optical density at OD_600_ reached 1.0. Subsequently, 1 mL of this culture was transferred into 100 mL of LB medium supplemented with L-tryptophan (0.2 mg/L) and then incubated at a temperature of 30 °C with continuous agitation at a speed of 180 rpm for a duration of 4 days, and then the supernatant was collected. Next, 2 mL of the supernatant was mixed with 2 mL of Salkowski reagent. After a dark incubation period of thirty minutes, the mixture was quickly analyzed using a spectrophotometer at OD_530_.

Salkowski reagent: 35% perchloric acid (50 mL), 0.50 mol·L^−1^ FeCl_3_ solution (1 mL).

Nitrogen fixation activity, phosphate solubilization activity, potassium dissolution activity, and siderophore production activity were tested on N-free Ashby medium, inorganic phosphorus medium, Alexandrov medium [40], and Chrome azurol S assay medium (CAS), respectively. Cbmb3 was grown in the different media at 35 °C for 7 days, then the growth of colonies on Ashby medium, the clear halos around bacterial colonies on inorganic phosphorus medium and Alexandrov medium, and the orange color change reactions on blue agar around bacterial colonies on CAS medium were observed, respectively.

N-free Ashby medium: mannitol 10 g/L, CaCO_3_ 5 g/L, KH_2_PO_4_ 0.2 g/L, MgSO_4_ 0.2 g/L, NaCl 0.2 g/L, CaSO_4_ 0.1 g/L, solidified with 2.5% agar.

Inorganic phosphorus medium: glucose 10 g/L, Ca(PO_4_)_2_ 5 g/L, (NH_4_)_2_SO_4_ 0.5 g/L, NaCl 0.3 g/L, KCl 0.3 g/L, MgSO_4_·7H_2_O 0.3 g/L, FeSO_4_·7H_2_O 0.003 g/L, MnSO_4_·4H_2_O 0.003 g/L, solidified with 2.5% agar.

Alexandrov medium: glucose 5 g/L, Na_2_HPO_4_ 2 g/L, potassium feldspar powder 1 g/L, MgSO_4_·7H_2_O 0.5 g/L, CaCO_3_ 0.1 g/L, FeCl_3_ 0.005 g /L, solidified with 2.5% agar.

CAS medium: Na_2_HPO_4_·12H_2_O 1.2 mg/L, NaH_2_PO_4_·2H_2_O 0.3 mg/L, HDTMA 0.07 mg/L, CAS 0.06 g/L, NH_4_Cl 0.12 mg/L, NaCl 0.06 mg/L, KH_2_PO_4_ 0.04 mg/L, FeCl·6H_2_O 0.003 mg/L, solidified with 2.5% agar.

## 3. Results

### 3.1. Isolation and Phenotypic Characteristics of Cbmb3

**Figure 1:**
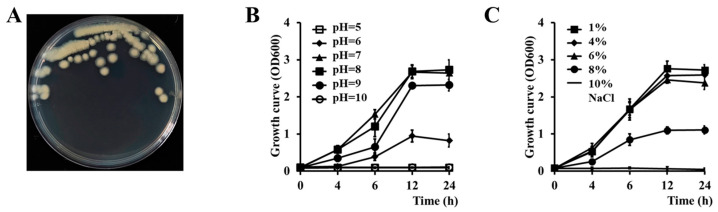
Phenotypic characteristics of the strain Cbmb3. (**A**) Morphological features of the colony. (**B**) Growth curves for different pH values. (**C**) Growth curves for different concentrations of NaCl.

The strain Cbmb3 was isolated from plastic-attached soil utilizing PVC as the sole carbon source. The colonies of the strain Cbmb3 grown on Luria–Bertani (LB) medium exhibit a light-yellow hue and possess a rounded morphology with irregular margins, as well as an obligate aerobic growth requirement (Figure 1A). The growth curve of the strain Cbmb3 in LB liquid culture medium is characterized by a brief lag phase, followed by exponential growth and then a stationary phase. Cbmb3 is capable of growing in environments with NaCl concentrations ranging from 1% to 8% and pH levels between 6.0 and 9.0 (Figure 1B,C).

### 3.2. Genomic Characteristics of Cbmb3

**Figure 2:**
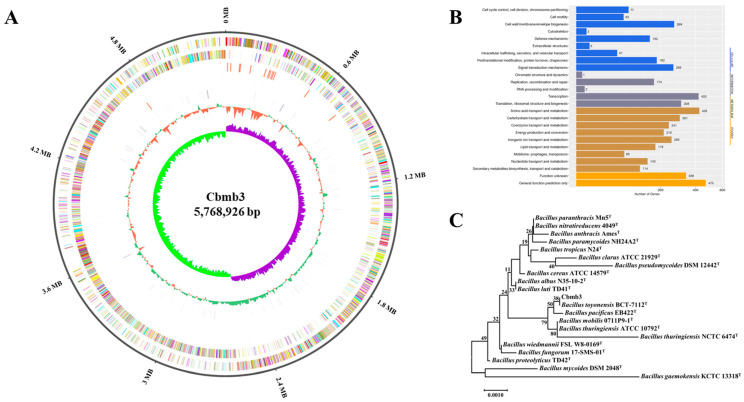
Genome features of the Cbmb3 chromosome. (**A**) A circular representation of the Cbmb3 chromosome. Rings represent the following features, labeled from outside to inside: ring 1, genome size; ring 2, forward-strand gene; ring 3, reverse-strand gene; ring 4, forward-strand ncRNA; ring 5, reverse-strand ncRNA; ring 6, repeat; ring 7, GC content; ring 8, GC skew, where green and purple correspond to above- and below-average GC skews, respectively. (**B**) The distribution of COG categories for chromosome genes. (**C**) The phylogenetic tree based on 16S rRNA of type strains (T).

The complete genome of Cbmb3 (Figure 2A) comprises a single circular chromosome and three plasmids (Figure S1). The size of the single circular chromosome is 5,768,926 bp, and the average guanine–cytosine (GC) content was 35.95%. The chromosome is composed of 5835 RAST-predicted coding sequences (CDSs) and 42 rRNA, 105 tRNA, and 36 sRNA genes (Table 1). The COG functional assignment revealed that 4009 CDSs (68.7%) were classified into 25 distinct COG categories; “Amino acid transport and metabolism” (425 CDSs), “Transcription” (420 CDSs), “Translation, ribosomal structure biogenesis” (309 CDSs), “Carbohydrate transport and metabolism” (301 CDSs), and “Cell wall/membrane/envelope biogenesis” (269) were the most enriched functional categories (Figure 2B). The distribution of functional elements is necessary to study the characteristics of the strain. In Cbmb3, 15 prophage regions were predicted by using PHAST, covering a 359.4 kb (6.23%) region of the chromosome (Table S1); 10 gene islands were identified by using IslandViewer4, covering a 195.4 kb (3.39%) region of the chromosome (Table S2); and 6 CRISPRs were identified by CRISPRCasFinder (Table S3).

### 3.3. Phylogenetic Analysis

The 16S rRNA sequence of Cbmb3 (1541 bp) was analyzed using EzBioCloud to search for similar species. A total of 20 16S rRNA sequences were collected and utilized to construct a phylogenetic tree utilizing the neighbor-joining method (Figure 2C, Table S4). The analysis revealed a 100% match with *Bacillus toyonensis* BCT-7112^T^ (accession number: CP006863). The Cbmb3 chromosome shared 98.47% ANI and 99.52% DDH values with other members of *Bacillus toyonensis* BCT-7112 ^T^ (NCBI accession number: PRJNA225857) (Table 2). Thus, the results indicated that Cbmb3 can be classified as a strain of *Bacillus toyonensis*.

### 3.4. PVC-Degrading Properties of Cbmb3

**Figure 3:**
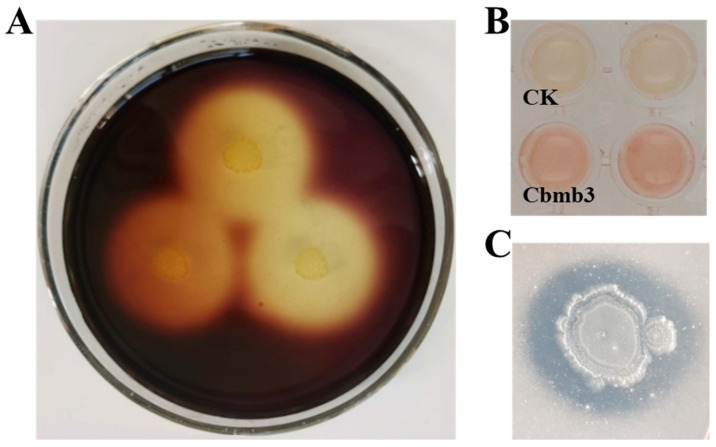
(**A**) PCV degradation properties of Cbmb3. (**B**) IAA secretion detection. (**C**) Phosphate solubilization activity.

The strain Cbmb3 exhibited the capability to grow on the MSM medium utilizing PVC as the sole carbon source and subsequently displayed clear zones surrounding colonies upon exposure to an iodine–boric acid solution (Figure 3A). The PVC film inoculated with the strain Cbmb3 exhibited a weight loss of 5.34% in the liquid biodegradation experiment.

The genome of the strain Cbmb3 was further analyzed to identify genes potentially involved in PVC biodegradation. Through KEGG metabolic pathway and network analyses, the strain Cbmb3 genome was found to encode some genes possibly involved in xenobiotic biodegradation and metabolism, such as chloroalkane and chloroalkene degradation or chlorocyclohexane and chlorobenzene degradation. The Cbmb3 genome was analyzed to identify genetic elements potentially associated with PVC degradation, and genes for several enzymes were found to play a putative role in biodegradation, including alcohol dehydrogenase, glutathione dehydrogenase, aldehyde dehydrogenase, haloacid dehalogenase, catalase–peroxidase, acetaldehyde dehydrogenase, and catechol 2,3-dioxygenase (Table 3). The catalase–peroxidase, aldehyde dehydrogenase, and catechol 2,3-dioxygenase would transform long-chain PVC into lower-molecular-weight polymers [10]. The haloacid dehalogenase might be involved in PVC dichlorination [41]. The different dehydrogenases might promote the production of -C=C- bonds and hydroxyl groups [42].

### 3.5. Plant-Growth-Promoting Properties of Cbmb3

The impact of MPs on soil quality and their potential negative effects on plants and microbiota have been a subject of controversy, with unresolved concerns regarding the hidden risks to soil environments and crop growth. Therefore, an assessment was conducted to explore the plant-growth-promoting capabilities of Cbmb3 to broaden its application scope. This assessment included evaluating its abilities in IAA production, nitrogen fixation, phosphorus solubilization, potassium dissolution, and siderophore production. The strain Cbmb3 exhibited capabilities for IAA synthesis and phosphorus solubilization (Figure 3B,C) while lacking the ability to carry out nitrogen fixation, potassium dissolution, and siderophore production.

Some genes associated with the promotion of plant growth were identified (Table 4). In Cbmb3, the synthesis of IAA occurs via the Trp-dependent pathway, which involves the *trpABFCDE* gene cluster responsible for tryptophan biosynthesis, a precursor for IAA production [43]. In terms of phosphate solubilization, the bacteria produce organic acids, primarily gluconic acid, to dissolve mineral phosphates. The synthesis of gluconic acid involves the glucose dehydrogenase enzyme encoded by *gcd* [44]. The *phn*-related operons (*phnCDEE*1) [45], the *pst* operon (*pstSCAB*) [46], and others were also identified in the chromosome of Cbmb3, which have been previously reported to be responsible for the uptake, degradation, and high-affinity free-phosphate transport system of phosphonate.

The strain’s capabilities to swim to waste plastics and colonize them are essential prerequisites for their degradation [47,48]. In the genome of the strain Cbmb3, a variety of genes related to colonization were identified (Table 4), such as a flagellar-associated operon (*flg-fli-flh*) [49,50,51], polysaccharide biosynthesis operon (*epsA-O*) [52], amyloid fiber biosynthesis operon (*tapA-sipW-tasA*) [53,54,55,56], autoinducer 2 (AI-2) synthesis gene (*luxS*, *lsr*), and chemotaxis-associated gene (*che*) [57,58], suggesting that Cbmb3 could be a potential strain to adsorb onto plastics and degrade them.

### 3.6. Antagonistic Activity of Cbmb3 against Plant Pathogens

**Figure 4:**
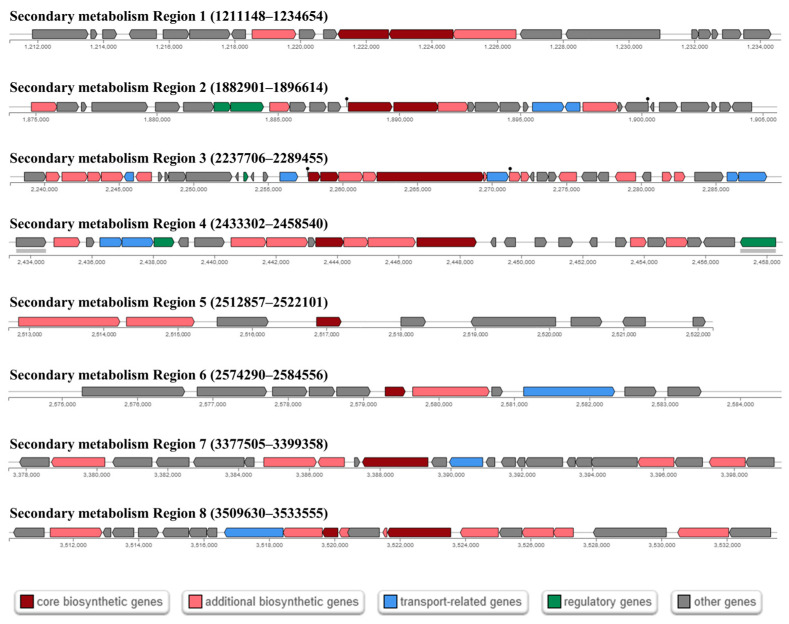
The genetic organization of gene clusters for secondary metabolism in the Cbmb3 chromosome.

The production of bioactive metabolites by soil microbes can suppress soilborne pathogen reproduction to promote plant growth [59,60]. The antiSMASH analysis identified eight putative biosynthesis gene clusters in the Cbmb3 genome (Figure 4; Table 5). Regions 2 and 8 showed 100% similarity to petrobactin and paeninodin. Region 3 was predicted to synthesize nonribosomal peptides (NRPs) and exhibited 85% similarity to bacillibactin. Region 4 was predicted to synthesize NRPs and exhibited 40% similarity to fengycin. Region 7 was predicted to synthesize a terpene. Region 1 was predicted to synthesize LAP (linear azol(in)e-containing peptides). Regions 5 and 6 were predicted to synthesize Ripps (other unspecified ribosomally synthesized and post-translationally modified peptide products). The regulation and biosynthesis of antimicrobial genes covered a 179,494 bp (3.14%) region of the whole genome, indicating that Cbmb3 has a certain capability against soilborne plant pathogens.

## 4. Discussion

The presence of microplastics in soil can compromise its structural integrity, alter the composition of soil aggregates and particle sizes, and affect porosity, consequently posing a threat to soil aeration and water retention capacity. Such alterations not only hinder the absorption of water and nutrients by crops but also potentially hinder their growth and development, leading to reduced crop yields and the degradation of farmland [61,62,63]. Meanwhile, recent research has indicated that microplastics have a significant impact on the presence of antibiotic resistance genes in soils, thereby facilitating the proliferation of these genes within agricultural environments [64,65]. Now, some microorganisms with PVC degradation activity have been reported, but the biodegradation mechanisms and the pathways remain largely unexplored. In the present work, we have isolated a PVC-degrading microorganism, *Bacillus toyonensis* Cbmb3, from plastic-attached farmland soil, and the whole-genome sequence was analyzed to enhance the understanding of PVC degradation.

The bacterium *Bacillus toyonensis* Cbmb3 demonstrated the ability to utilize PVC as a carbon source, so we searched for potential genes associated with the biodegradation of PVC via microbial genome analysis. Several genes, including alcohol dehydrogenase, glutathione dehydrogenase, aldehyde dehydrogenase, haloacid dehalogenase, acetaldehyde dehydrogenase, and catechol 2,3-dioxygenase, were identified as potentially involved in the biodegradation of PVC [10]. However, genes encoding other key enzymes, such as laccase [66] or monooxygenase [67], were not detected. In future investigations, our point research object will focus on the metabolic processes of PVC degradation, and gene knockout technology, transcriptome analyses, metabolite analyses, and GC-MS will be employed to explore the metabolic processes of PVC degradation in *Bacillus toyonensis* Cbmb3, aiming to unveil additional involved genes and intermediate metabolites while completing the proposed pathway.

The presence of microplastics in agricultural soil can lead to alterations in soil physicochemical properties and biodiversity and may result in the accumulation of microplastics in crops through uptake, posing a potential threat to food safety [2,7]. Given the potential risks associated with microplastics, it is imperative to develop strategies aimed at reducing their presence in agricultural soils. Some bacteria capable of utilizing microplastics have been applied as agricultural bioremediators, facilitating the remediation of environmental plastics in agricultural settings, particularly plant-growth-promoting bacteria (PGPB) [9,14]. PGPB encompass a diverse array of microorganisms that facilitate plant growth through various mechanisms, including alleviating abiotic stresses, synthesizing phytohormones, fixing nitrogen, and suppressing pathogenic fungi [68,69,70]. The assays used to explore the presence of plant-growth-promoting capabilities and the associated gene analysis revealed that *Bacillus toyonensis* Cbmb3 exhibits promising potential as a strain for developing effective bioremediators; however, its application effects on crops have not been tested. Therefore, pot experiments will be employed in future studies to investigate the role of Cbmb3 in enhancing plant stress resistance against PVC. Once optimized, Cbmb3 could aid in the removal of accumulated PVC while, at the same time, providing the co-benefit of promoting plant growth in remediated agricultural lands.

## 5. Conclusions

Our study presents a PVC-degrading microorganism isolated from a soil sample attached to waste plastic; this report represents the first whole-genome sequence analysis of a *Bacillus* strain with PVC-degrading properties. The phylogenetic analysis, combining 16s rRNA and genome data with ANI and in silico DDH values, provides the taxonomic position of Cbmb3 in the genus *Bacillus toyonensis*. Cbmb3 exhibited PVC-degrading capabilities on the screening agar medium by utilizing PVC as the sole carbon source, as evidenced by the formation of clear zones around the colony upon flooding with an iodine–boric acid solution. Moreover, we have identified putative PVC-degrading genes in the chromosome of Cbmb3, providing a genetic basis for the degradation of PVC by Cbmb3. Experiments on the presence of activity or compounds promoting plant growth revealed the potential of Cbmb3 in producing IAA and solubilizing phosphorus, while a systematic analysis of the Cbmb3 chromosome identified the genetic basis underlying its contributions to plant growth promotion and biocontrol. Overall, this study presents the first complete genome of *Bacillus toyonensis* with PVC-degrading properties, offering comprehensive insights into the genomic characteristics of this species and enhancing our understanding of microbial PVC degradation. 

## Figures and Tables

**Table 1:** General chromosomal features of Cbmb3.

Feature	Cbmb3
Genome (bp)	5,768,926 bp
C + G (%)	35.95%
CDS number	5835
rRNA	42
tRNA	105
sRNA	36
Tandem Repeat	405
Prophage region	15
Gene island	10
CRISPR	6
Carbohydrate-Active enZYmes	148
Genes assigned to GO	3163
Genes assigned to COG	4009
Genes assigned to KEGG	3017

**Table 2:** Comparative genomic analysis of Cbmb3 with *Bacillus* genomes.

Strains	Accession No.	ANI (%)	dDDH (%)	Size (Mp)	G + C (%)
Cbmb3	PRJNA992983	100.00	100.00	5.76	35.46
*Bacillus toyonensis* BCT-7112	PRJNA225857	98.47	99.52	4.94	33.78
*Bacillus toyonensis* BPN45/4	PRJNA521676	98.43	99.25	5.45	35.20
*Bacillus toyonensis* P18	PRJNA678769	98.53	98.92	5.25	35.19
*Bacillus wiedmannii* SR52	PRJNA490767	91.31	60.98	5.45	35.46
*Bacillus luti* FJ	PRJNA515150	91.07	54.84	5.20	35.71
*Bacillus albus* SXL388	PRJNA960711	90.90	47.45	5.35	35.25
*Bacillus mobilis* 16-00177	PRJEB20065	90.79	34.26	5.69	35.28
*Bacillus thuringiensis* ATCC 10792	PRJNA29723	91.44	29.40	6.26	34.82
*Bacillus mobilis* 0711P9-1	PRJNA325888	90.75	27.99	5.66	35.30
*Bacillus proteolyticus* TD42	PRJNA325897	90.60	24.19	5.86	35.15
*Bacillus pacificus* anQ-h4	PRJNA762228	90.81	22.98	5.25	35.41
*Bacillus fungorum* 17-SMS-01	PRJNA408208	91.13	9.29	5.65	34.99

**Table 3:** Genes associated with PVC degradation in the Cbmb3 genome.

Enzymes	Orthologous Genes	Locus Tags
Alcohol dehydrogenase [EC:1.1.1.1]	K00001, K13953	02186, 02529, 03409, 02226
Glutathione dehydrogenase [EC:1.1.1.284]	K00121	03056
Aldehyde dehydrogenase [EC:1.2.1.3]	K00128	01296, 02750, 03453
Haloacid dehalogenase [EC:3.8.1.2]	K01560	03232, 05362
Catalase-peroxidase [EC: 1.11.1.6]	K03781	01144, 04758
Acetaldehyde dehydrogenase [EC:1.2.1.10]	K04072	04341
Catechol 2,3-dioxygenase [EC:1.13.11.2]	K07104	03431, 04330

**Table 4:** Plant-growth-promotion-associated genes identified in Cbmb3 genome.

Plant Growth Promotion Traits	Gene ID	Gene Name	Function
Indole-3-acetic acid (IAA)	01174, 03242	*trpS*	Tryptophan-tRNA ligase
	01229–01235	*trpABFCDE*	Tryptophan biosynthesis operon
Phosphonate solubilization	03367, 04700	*gcd*	Glucose dehydrogenase
	03582–03585	*phnCDEE1*	Phosphonate ABC transporter permease
	04241	*phoU*	Phosphate transport system regulatory protein
	04242–04244	*pstCAB*	Phosphate ABC transporter
Biofilm	01288–01290	*tapA-sipW-tasA*	Amyloid fiber biosynthesis protein
	03614–03600	*epsA-O*	Polysaccharide biosynthesis protein
	03708–03710	*pgsABC*	γ-poly-glutamate biosynthesis protein
	04852–04856	*glgPADCB*	Glycogen biosynthesis
Chemotaxis and motility	01638–01643	*che* operon	Chemotaxis-associated protein
	01648–01679	*flg-fli-flh* operon	Flagellar-associated operon
	00419, 00573, 00591, 01956, 01956, 04999	*mcpAC*	Methyl-accepting chemotaxis protein
Quorum sensing	04778	*luxS*	Autoinducer 2 (AI-2) synthesis protein
	02889–02894	*lsrBDCA-RK*	AI-2 uptake operon and transcriptional regulator

**Table 5:** Clusters of bioactive metabolite synthesized by Cbmb3 identified by antiSMASH.

Region	Type	Cluster Position	Metabolites	MIBiG ID	Similarity
1	LAP	1211148–1234654	-	-	-
2	NI-siderophore	1882901–1896614	Petrobactin	BGC0000942	100%
3	NRP	2237706–2289455	Bacillibactin	BGC0000309	85%
4	NRP	2433302–2458540	Fengycin	BGC0001095	40%
5	Ripp	2512857–2522101	-	-	-
6	Ripp	2574290–2584556	-	-	-
7	terpene	3377505–3399358	Molybdenum cofactor	BGC0000916	17%
8	lassopeptide	3509630–3533555	Paeninodin	BGC0001356	100%

## Data Availability

Data are available upon request due to privacy and ethical restrictions.

## Supplementary Materials

The following supporting information can be downloaded at https://www.mdpi.com/article/10.3390/jox14010018/s1. Table S1: The features of prophages in the Cbmb3 genome. Table S2: The features of genomic islands in the Cbmb3 genome. Table S3: The features of CRISPR in the Cbmb3 genome. Table S4: Closely related species of Cbmb3 based on EzBioCloud. Figure S1: The structure and functional analysis of the three plasmids of Cbmb3. Rings represent the following features, labeled from outside to inside: ring 1, genome size; ring 2, forward-strand gene; ring 3, reverse-strand gene; ring 4, GC content; ring 5, GC skew, where green and purple correspond to above- and below-average GC skews, respectively.
