# Supplementary material for: The Complete Genome Sequence of Bacillus toyonensis Cbmb3 with Polyvinyl Chloride-Degrading Properties
Source: J Xenobiot. 2024 Feb 26;14(1):295–307. doi: 10.3390/jox14010018 (PMC10970875; doi:10.3390/jox14010018)
Supplement: Supplementary file 1 [file jox-14-00018-s001.zip › jox-2781306-supplementary.pdf]

Journal of Xenobiotics

Supplementary Materials: The Complete Genome Sequence of *Bacillus toyonensis* Cbmb3 with Polyvinyl Chloride-Degrading Properties

Dandan Wang, Hong Yu, Xinbei Liu, Li Sun, Xijian Liu, Ruilong Hu, Chao Wang, Yuping Zhuge and Zhihong Xie

Table S1. The features of prophages in the Cbmb3 genome.

Phage	Sequence	Phage Start	Phage End	attL Star	attL End	attR Start	attR End
pp1	Chromosome	50173	63344	51058	51071	62210	62223
pp2	Chromosome	94067	149447	96527	96540	148622	148635
pp3	Chromosome	922511	933435	925776	925819	930325	930368
pp4	Chromosome	1599210	1617030	1600191	1600204	1630124	1630137
pp5	Chromosome	2061457	2097658	2061979	2061994	2094320	2094335
pp6	Chromosome	2890585	2906656	2891107	2891122	2923448	2923463
pp7	Chromosome	3520140	3551592	3520662	3520677	3553003	3553018
pp8	Chromosome	3700378	3723515	3700900	3700915	3733241	3733256
pp9	Chromosome	4038837	4070854	4038729	4038745	4068435	4068451
pp10	Chromosome	4094198	4107464	4092358	4092448	4107762	4107852
pp11	Chromosome	4168832	4190612	4168724	4168740	4198430	4198446
pp12	Chromosome	4445220	4453837	4444340	4444487	4451052	4451199
pp13	Chromosome	4808717	4824417	4806877	4806967	4822281	4822371
pp14	Chromosome	5064541	5118750	5066218	5066232	5117666	5117680
pp15	Chromosome	5286012	5295672	5285132	5285279	5291844	5291991
pp16	Plasmid1	86459	160039	84820	84834	159638	159652
pp17	Plasmid2	477	32466	973	994	35384	35407
pp18	Plasmid3	113	11616	1121	1268	7833	7980
pp19	Plasmid3	63806	70514	62926	63073	69638	69785

Table S2. The features of genomic islands in the Cbmb3 genome.

Genomic island	Start	End	Region length (bp)
GI-1	919,795	955,858	36,063
GI-2	1,234,820	1,262,831	28,011
GI-3	2,045,356	2,070,545	25,189
GI-4	3,341,575	3,351,698	10,123
GI-5	3,557,657	3,567,416	9,759
GI-6	3,669,313	3,696,736	27,423
GI-7	3,746,391	3,757,251	10,860
GI-8	4,035,589	4,050,440	14,851
GI-9	4,107,631	4,111,703	4,072
GI-10	4,261,446	4,290,478	29,032

Table S3. The features of CRISPR in the Cbmb3 genome.

CRISPR	Sequence	CRISPR Start	CRISPR End	CRISPR Length (bp)
**1**	**Chromosome**	**1070099**	**1070185**	**86**
**2**	**Chromosome**	**1070531**	**1070674**	**143**
**3**	**Chromosome**	**1070912**	**1071054**	**142**
**4**	**Chromosome**	**4821392**	**4821500**	**108**
**5**	**Chromosome**	**4874331**	**4874438**	**107**
**6**	**Chromosome**	**5080856**	**5080989**	**133**
**7**	**Plasmid**	**164870**	**164947**	**77**
**8**	**Plasmid**	**72978**	**73401**	**423**
**9**	**Plasmid**	**36826**	**37785**	**959**
**10**	**Plasmid**	**37883**	**38113**	**230**

Table S4. Closely related species of Cbmb3 based on EzBioCloud.

Strains	Accession	Similarity
Bacillus toyonensis **BCT-7112**	CP006863	100
Bacillus mobilis **0711P9-1**	MACF01000036	99.93215739
Bacillus pacificus **EB422**	KJ812450	99.93215739
Bacillus thuringiensis **ATCC 10792**	ACNF01000156	99.93215739
Bacillus wiedmannii **FSL W8-0169**	LOBC01000053	99.79647218
Bacillus proteolyticus **TD42**	MACH01000033	99.72862958
Bacillus albus **N35-10-2**	MAOE01000087	99.72862958
Bacillus luti **TD41**	MACI01000041	99.72862958
Bacillus fungorum **17-SMS-01**	MG601116	99.72862958
Bacillus cereus **ATCC 14579**	AE016877	99.66078697
Bacillus paramycoides **NH24A2**	MAOI01000012	99.66078697
Bacillus tropicus **N24**	MACG01000025	99.66078697
Bacillus paranthracis **Mn5**	MACE01000012	99.59294437
Bacillus nitratireducens **4049**	KJ812430	99.59294437
Bacillus anthracis **Ames**	AE016879	99.52510176
Bacillus clarus **ATCC 21929**	MH918154	99.45725916
Bacillus mycoides **DSM 2048**	ACMU01000002	99.38941655
Bacillus thuringiensis **NCTC 6474**	UAPX01000031	99.37759336
Bacillus pseudomycoides **DSM 12442**	ACMX01000133	99.25373134
Bacillus gaemokensis **KCTC 13318**	LTAQ01000012	98.9137814

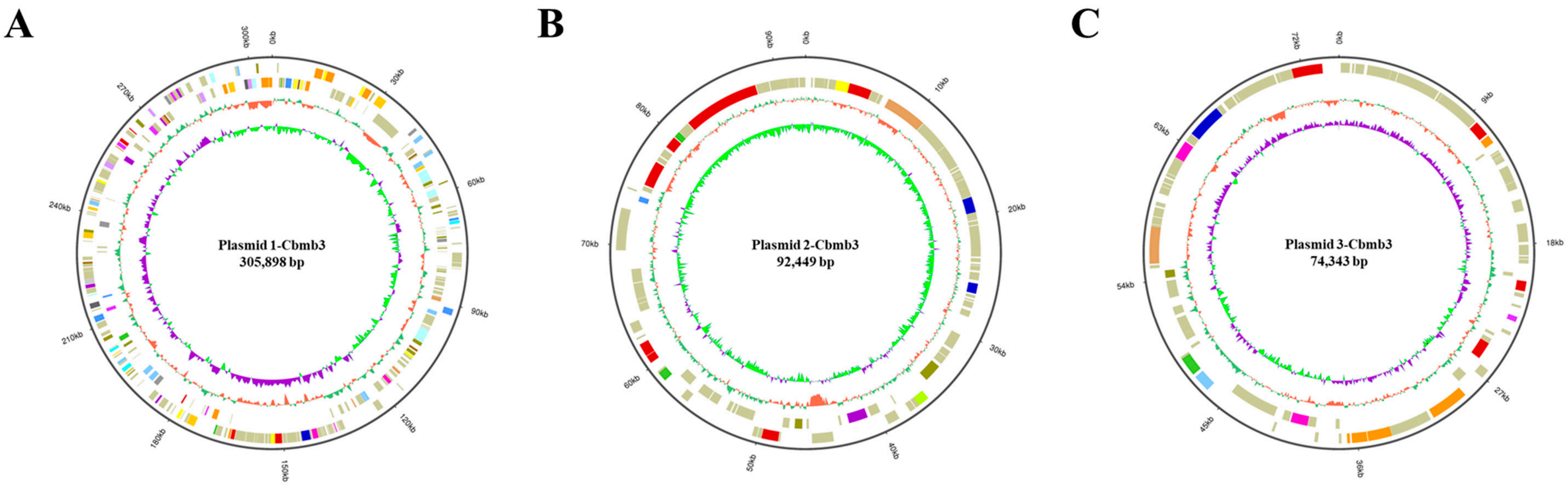

Figure S1. Structure and functional analysis of the three plasmids of Cbmb3. Rings represent the following features labeled from outs ide to inside: ring 1, genome size; ring 2, forward strand gene; ring 3, reverse strand gene; rings 4, GC content; ring 5, GC-skew, green and purple correspond to above- and below-average GC skew, respectively.
